# Quantification of the tissue-culture induced variation in barley (*Hordeum vulgare *L.)

**DOI:** 10.1186/1471-2229-7-10

**Published:** 2007-03-02

**Authors:** Piotr T Bednarek, Renata Orłowska, Robert MD Koebner, Janusz Zimny

**Affiliations:** 1 Plant Breeding & Acclimatization Institute, 05-870 Błonie, Radzików, Poland; 2 UWM, Olsztyn, Poland; 3 John Innes Centre, Colney Lane, Norwich NR4 7UH, UK

## Abstract

**Background:**

When plant tissue is passaged through *in vitro *culture, many regenerated plants appear to be no longer clonal copies of their donor genotype. Among the factors that affect this so-called tissue culture induced variation are explant genotype, explant tissue origin, medium composition, and the length of time in culture. Variation is understood to be generated via a combination of genetic and/or epigenetic changes. A lack of any phenotypic variation between regenerants does not necessarily imply a concomitant lack of genetic (or epigenetic) change, and it is therefore of interest to assay the outcomes of tissue culture at the genotypic level.

**Results:**

A variant of methylation sensitive AFLP, based on the isoschizomeric combinations *Acc65*I/*Mse*I and *Kpn*I/*Mse*I was applied to analyze, at both the sequence and methylation levels, the outcomes of regeneration from tissue culture in barley. Both sequence mutation and alteration in methylation pattern were detected. Two sets of regenerants from each of five DH donor lines were compared. One set was derived via androgenesis, and the other via somatic embryogenesis, developed from immature embryos. These comparisons delivered a quantitative assessment of the various types of somaclonal variation induced. The average level of variation was 6%, of which almost 1.7% could be accounted for by nucleotide mutation, and the remainder by changes in methylation state. The nucleotide mutation rates and the rate of epimutations were substantially similar between the andro- and embryo-derived sets of regenerants across all the donors.

**Conclusion:**

We have developed an AFLP based approach that is capable of describing the qualitative and quantitative characteristics of the tissue culture-induced variation. We believe that this approach will find particular value in the study of patterns of inheritance of somaclonal variation, since non-heritable variation is of little interest for the improvement of plant species which are sexually propagated. Of significant biological interest is the conclusion that the mode of regeneration has no significant effect on the balance between sequence and methylation state change induced by the tissue culture process.

## Background

When plant tissue is passaged through *in vitro *culture, many regenerated plants appear to be no longer clonal copies of their donor genotype. Among the factors that affect this so-called tissue culture induced variation are explant genotype, explant tissue origin, medium composition, and the length of time in culture. Variation is understood to be generated via a combination of genetic and/or epigenetic changes, and although it is generally considered to be detrimental in the context of plant improvement, in some situations can nevertheless be exploited to good effect. A lack of any phenotypic variation between regenerants does not necessarily imply a concomitant lack of genetic (or epigenetic) change, and it is therefore of interest to assay the outcomes of tissue culture at the genotypic level. DNA-based marker systems can detect some of these changes, but most currently available platforms focus on genetic, rather than on epigenetic mutation [1,2]. A few do detect variation in patterns of DNA methylation [3,4], but those able to simultaneously evaluate sequence and methylation changes are rare [5-7]. Since these techniques are, by and large, insufficiently sensitive to expose more than a sample of the full range of somaclonal variation [8], it is of interest to develop a focused marker platform specifically targeted to assay this type of variation.

We argue here that an AFLP-based method, which exploits a pair of isoschizomers which differ in their sensitivity to recognition site methylation, provides an effective means to quantify the characteristics of tissue culture induced variation. We have used this approach to characterise induced variation in barley (*Hordeum vulgare*), by comparing outcomes following *in vitro *culture following the induction of either embryogenesis (immature embryo explant) or androgenesis (microspores). This has allowed us to determine whether explant identity influences the frequency or type of variation induced, and to identify which, if either, of these types predominate.

## Results

In all, 78 independent plants were regenerated from tissue culture, with comparable representation between the embryogenic- ('E') and androgenic- ('A') derived sets (Table 1). All regenerants were fully self-fertile, and were indistinguishable in phenotype from their donor plant. Reproducible metAFLP fingerprints were obtained both from the donors and regenerants. The fingerprints of the five donor parents, along with those of 11 further sib individuals, with respect to over 750 AFLP fragments (ten primer combinations), showed an almost complete (> 99.7%) level of uniformity, demonstrating that AFLP profiles are extremely consistent between sexually produced sib individuals. The numbers of metAFLP fragments scored in the regenerants were 323 from the 8DH donor/regenerant group, 332 for 5DH, and 412 for 4DH, 9DH and 16DH. About 93% of these fragments were unaffected by tissue culture. The absolute frequencies of each altered AFLP pattern (reflecting putative sequence or methylation pattern changes at, or in the vicinity of the restriction endonuclease recognition site(s)) are given in Table 2. For ease of reference, these have each been assigned a binary code, as explained in Methods (Data scoring and analysis). Of note is that all but two of the set of possible codes were recovered among the regenerants. No single regenerant was free of (epi)mutations, with a mean of about 29 alterations per plant (range 12 to 58) being detected. The number of these alterations per individual 'E' regenerant varied from 13 to 46, and per 'A' regenerant from 14 to 64.

For the 'E' regenerants, the explant tissue is diploid and assumed to be fully homozygous, since it was derived from the first generation progeny of a doubled haploid individual (itself having been generated from a single isolated microspore). For these plants, therefore, the expectation was that tissue culture-induced events would be restricted to the gain of AFLP fragments, as the loss of a donor fragment requires that both homologues be simultaneously (epi)mutated. In contrast, for the 'A' regenerants, which are largely haploid during their passage through tissue culture, both fragment loss and gain are equally possible. However, we were surprised to observe the frequent occurrence of fragment loss among the 'E' regenerants (codes 10** and **10). Although we have no convincing biological explanation for these events, it is clear from much of the literature surrounding somaclonal variation that tissue culture regenerants frequently suffer from phenotypic abnormalities [9] including known recessive phenotypes [10]. Moreover, a substantial proportion of methylation changes detected in maize regenerants were found to be in the homozygous state [11].

We used ANOVA to examine whether the frequency of tissue-culture induced changes was affected by either the identity of the explant, or of the doubled haploid sib donor (Table 3). This established that the identity of the explant has no influence over the frequency of tissue-culture induced variation. Surprisingly, there does appear to be a significant effect of donor genotype.

**Figure 1:**
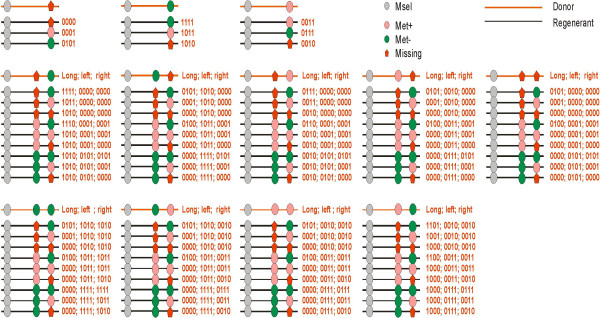
A schematic to interpret the outcomes on regenerant AFLP profiles of (epi)mutation events induced during tissue culture. The possible bases of AFLP profile changes induced during tissue culture are shown in terms of methylation of one or more sites and point mutation at the enzyme recognition site(s). All possible patterns were assigned a binary code. Top row: A single KpnI/*Acc65*I recognition site within the resolvable amplicon; lower two rows: a pair of KpnI/*Acc65*I sites within the resolvable amplicon. Grey circle – represents *Mse*I site; pink circle – methylated site digested by *Kpn*I but not *Acc65*I; green circle – non-methylated site digested by *Kpn*I and *Acc65*I; red pentagon – former *Acc65*I/*Kpn*I after mutation; red and black lines – donor and regenerant DNAs, respectively.

Based on the most parsinomious explanation (i.e., the minimum number of (epi)mutations) for each 1-0 pattern (Figure 1), we have grouped (epi)mutations of the same type into four types: sequence variation, demethylation, *de novo *methylation and complex events. The sequence variation group was made up from (0001), (0010), (0101), (1010), (1110) and (1101); demethylation by (0111); *de novo *methylation by (1011); and complex events by (0100), (1001), (0110) and (1000). We tested whether any of these groups contributed significantly more (or less) to the overall level of variation, and found little evidence to support a bias towards any specific variation type. However, it does appear that methylation events, taken as a whole, (i.e., both *de novo *methylation and demethylation, along with some of the 'complex' events, which must include an element of methylation/demethylation and sequence change), account for at least one half of the tissue-culture induced changes.

A calculation of the average value of sequence and methylation changes for both 'E' and 'A' sets for all donor-regenerant sets showed that the frequencies of sequence changes were 1.46% and 1.90%, respectively. Similarly, the frequencies of alteration in methylation patterns were 3.38% and 3.57%. Although differences obtained between the 'E' and 'A' sets between these frequencies, none were of any statistical significance.

Certain fragments appeared to behave non-randomly in response to tissue culture, in the sense that the profile of 90–100% of the regenerants from a given donor included a novel metAFLP fragment absent from the profile of the donor (or *vice versa*). A summary of the patterns that behaved in this way is given in Table 4. The most common such patterns are (0111) (demethylation), (1011) (*de novo *methylation) and (1000) (complex event).

## Discussion

The metAFLP assay is very stable, and is therefore well suited as a platform for the detection of induced mutation. Tissue culture clearly induces a much higher rate of (epi)mutation than the background rate occurring during sexual reproduction. The latter is estimated to be of the order of 0.04% for *Acc65*I/*Mse*I and 0.03% for *Kpn*I/*Mse*I fragments, which is about two orders of magnitude lower than the estimated frequencies of somaclonal variation. Previous demonstrations that methylation patterns can be altered by the tissue culture process have been provided by a number of studies [11-14], and at least some induced epimutations have been shown to be sexually transmissible [11]. The approach described here extends these observations by providing a quantitative picture of what occurs at the DNA level and at the level of its methylation. Most importantly, the large discrepancy in metAFLP variation between the sexually and asexually derived progenies establishes that the majority of the (epi)mutations present in the latter must have arisen as a consequence of the tissue culture process.

The statistical indication that the identity of the donor does influence the frequency of mutation induction is surprising, as each donor individual was a selfed progeny of a fully homozygous (doubled haploid) plant, so that they must be, to all intents and purposes, genetically identical to one another. It is unlikely that variation in environmental factors (specifically, variation in the conditions of tissue culture) can explain this unexpected result, since all the regenerants were prepared from a single experimental batch. It is possible therefore that the significantly different frequencies of events per regenerant (for 4DH, only 12 regenerants were analysed) and the modest number of regenerants per donor within each set has produced a false positive result, which would be expected to disappear once either more progeny per set and/or more progeny sets were analysed. Alternatively, the "donor" effect may be a statistical artefact of the Tukey-Kramer test which, when used to contrast variable numbers of regenerants within a data set, compares the sets in pairs, and thus is capable of delivering a non-existent "genotype" effect. Thus at present we lack a satisfactory explanation for the apparent donor effect.

Of significant biological interest is the conclusion from the adjusted analysis that the mode of regeneration has no significant effect on the balance between sequence and methylation state change induced by the tissue culture process. This finding contradicts the commonly held belief that plants regenerated without a passage through a callus phase are less prone to somaclonal variation than those emerging after a period in callus [15-17]. This view has generated a widespread preference for anther culture over embryo culture for both micropropagation and for the generation of doubled haploid progenies, widely used in both breeding and genetic analysis. In cereals at least, we suggest that this preference rests on a weak evidential foundation. Furthermore, the level of (epi)mutation affecting the 'A' set of regenerants implies that the interpretation of phenotype in doubled haploid mapping populations, which are ubiquitously used for genetic analysis of QTL, is not as straightforward as is generally assumed. A common experience in most QTL mapping projects is that considerably less than 100% of the phenotypic variation can be assigned to a set of identified QTL, and the common conclusion is that the unaccounted for fraction is under the control of many genes, each being responsible for too small an effect to be individually detected. On the basis of the present data, we would suggest that a significant proportion of the 'unexplained' variation manifested in these populations is in fact of somaclonal origin, and thus cannot be assigned to allelic variation distinguishing the parents of a mapping population.

It has been widely assumed that it is the disorganized state of the callus phase which is responsible for the release of cellular control over mutagenesis, whether generated by exogenous (UV, stress etc.) or endogenous (primarily transposons) agents, and/or that such tissue is compromised in the repair of somatic replication error. However, it appears that this model is unlikely to be correct, at least in barley, unless the process of androgenesis includes a short period of callus-like growth, which is not experimentally observable. Even if this is the case, however, the callus phase must be very short, and according to Jähne and Lörz (1995) [18] a short regeneration time diminishes the risk of variation. It is particularly interesting that the (epi)mutation rates in the 'E' and 'A' sets are so similar, because the former is a diploid tissue, which is likely to enjoy a higher capability for physiological buffering than the haploid tissue present during most of the androgenesis process.

Our estimates for the level of the tissue culture induced variation and its distribution between mutation and epimutation are largely consistent with those for bread wheat (*Triticum aestivum*) [19], in which a wide range of variation was associated with chromosomal changes, and with those for *H. bulbosum *[20], in which DNA methylation changes were detected. In contrast, the frequency of *Eco*RI/*Mse*I AFLP among doubled haploid wheat lines derived via androgenesis was less than 0.3% [21]. It is unclear whether this low level is characteristic of wheat (as opposed to barley), or whether it is connected with either the polyploid state, and/or with the restriction enzyme combination employed, since many of those commonly used are less able to expose intra-specific polymorphism than can *Pst*I/*Mse*I or certain other combinations [13,16,22,23].

Suggestions have been made that somaclonal variants tend to be concentrated in "hotspots". Thus, for example, heavy metal stress has been shown to direct DNA methylation to specific loci [24], while hypervariable RAPD products have been observed among the tissue culture regenerants of rye [25]. An indication of the existence of non-randomly behaving fragments in the present materials comes from the observation that some of the possible metAFLP patterns (0001, 1001, 0010, 0110) were not identified in the present experiments (either among the embryo- or the anther-derived regenerants) and the others (0101, 1110) were very rare, providing circumstantial evidence that some events are largely, or completely prohibited. Larger progeny sets will be necessary to establish whether the former type of event is rare as opposed to its being disallowed. Supporting the notion of non-random induction of (epi)mutations in tissue culture is the observation that a number of the demethylation and *de novo *methylation events, as well as sequence changes, were inherited by all (or at least 90%) of the regenerants, whether from the 'A' or the 'E' set. Interestingly, most of these non-random events affected methylation status, rather than being sequence changes.

## Conclusion

In summary, we have developed an AFLP based approach that is capable of describing the qualitative and quantitative characteristics of tissue culture-induced variation. We believe that this approach will find particular value in the study of patterns of inheritance of somaclonal variation, since non-heritable variation is of little interest for the improvement of plant species which are sexually propagated. Of more basic interest is to develop a mechanistic understanding of the somaclonal (epi)mutation process, since this may lead to the possibility of its manipulation, for example by the modification of the tissue culture medium. If the generally held understanding is correct that epigenetic control is exerted largely via DNA methylation, then the possibility arises that traits under epigenetic control could be marked by epi-alleles, and the metAFLP assay would have the potential to derive and evaluate markers for such alleles.

## Methods

### Plant material

Five full sib progeny of a single doubled haploid barley plant, obtained via androgenesis from isolated microspores [26], were used as donors of immature embryos and microspores. The donor plants were grown under controlled conditions (16 h/8 h day/night regime, light intensity 27 μmolm^-2^s^-1 ^and day/night temperatures of, respectively, 10°C and 12°C). Somatic embryogenesis was induced from immature embryo-derived calli [27] grown for three weeks on a semi-solid N6 medium [28] containing 2 mgl^-1 ^2,4-D. In order to encourage germination, somatic embryos were excised from callus, and transferred to 190–2 regeneration medium [29] containing 0.4 μM kinetin. Emerging plantlets were transferred to Erlenmeyer flasks on half-strength MS medium without growth hormones until they had reached 10–12 cm in height, after which they were potted in soil, acclimatized for two weeks, and finally grown to maturity under standard greenhouse conditions. To induce androgenesis, tillers were harvested when the microspores were between the uni- and the bi-nucleate stage. The cut ends of tillers were maintained in water under low light intensity for seven days at 4°C. The spikes were then surface sterilized by dipping in 70% ethanol followed by 10% sodium hypochloride, after which they were rinsed five times in sterile water. Anthers were excised, placed on semi-solid N6 medium [28] supplemented with 2 mgl^-1 ^2,4-D and 0.5 μM kinetin, and cultured in the dark at 26°C. Embryogenic calli were transferred to 190–2 regeneration medium [29]. Developed androgenic embryos were transferred to fresh 190–2 medium [30] solidified with 5 g/l Phytagel and supplemented with hormones [29], where they were kept under a 16 h/8 h day/night regime. Plantlets were rooted in glass tubes, and later potted into soil, transferred to a growth chamber for a short acclimatization period, and grown to maturity in a controlled environment chamber. Before anthesis, spikes were bagged to ensure self-pollination.

The primary regenerant plants, obtained via somatic embryogenesis (Set 'E'), and via androgenesis (Set 'A'), along with the five full sib donor plants (encoded 4DH, 5DH, 8DH, 9DH and 16DH, Table 1) formed the base material to study tissue culture-induced variation.

### DNA manipulation

Total genomic DNA was isolated from about 100 mg of 7-day-old seedling leaf, using the Dneasy MiniPrep kit (Qiagen). Oligonucleotides for metAFLP (Table 5) were obtained from the Centre for Macromolecular Studies of the Polish Academy of Sciences (Łódź). The AFLP procedure followed standard methods [31] with some minor modifications [32], using either *Acc65*I/*Mse*I or *Kpn*I/*Mse*I for the initial digestion of genomic DNA. *Acc65*I and *Kpn*I are isoschizomers, which differ in their sensitivity to template methylation. The former is insensitive to *dam *methylation, but its activity is blocked by both *dcm *and CpG methylation, while the latter is insensitive to all forms of methylation. About 500 ng of DNA were digested at 37°C for 3 h, stopping the reaction with a 70°C incubation for 15 min. The relevant adaptors were ligated to the restriction digest in a 25 μl volume at 20°C for 12 h and diluted 1:3 in TE buffer. Non-selective PCR was conducted with pre-selective primers in 25 μl reactions, using a standard amplification profile. The products of the pre-selective reaction were diluted 1:20 in TE, and selective 10 μl PCRs were carried out in the presence of 5'-(^32^P) labeled selective primers. Seven selective primer combinations (CpXpG-TGC/MCCG, CpG-GAC/MCCA, CpG-GCA/MCGC, CpG-GGC/MCAA, CpXpG-AGG/CAG, CpXpG-AGA/MCAA, CpXpG-AGC/MCCA) were applied to the regenerants from all four donors, CpXpG-AGG/MCAC to those from DH4, 8 and 16, and CpG-GAC/MCAA to those from DH4 and 16. Post PCR, samples were denatured with 6 μl of 80% formamide loading buffer in the presence of bromophenol blue and xylene cyanol. After further denaturation (95°C for 10 min followed by quenching at 5°C), 6 μl of the reaction mix were electrophoresed in 7% 50 cm × 0.4 mm denaturing polyacrylamide gels, and finally the gels were exposed to X-ray film overnight at -70°C.

### Data scoring and analysis

AFLP profiles were recorded as 1/0 binary matrices, where 1 indicates the presence and 0 the absence of a given fragment. The coding for each individual thus describes the presence/absence of each fragment in, respectively, the *Acc65*I/*Mse*I digests of the donor and a given regenerant, followed by the *Kpn*I/*Mse*I digests of the same donor and regenerant. Three of these codes relate to fragments unaffected by tissue culture: (1111) represents a fragment present in both digests of both donor and regenerant; (0011) a fragment present in the *Kpn*I/*Mse*I but not the *Acc65*I/*Mse*I digests of both donor and regenerant, which results from the situation where methylation at the recognition site prevents its digestion by *Acc65*I, but not by *Kpn*I; and (1100) a fragment present in *Acc65*I/*Mse*I, but not *Kpn*I/*Mse*I digests of both donor and regenerant – these can be formed where two adjacent *Acc65*I/*Kpn*I sites exist, one of which is methylated, but the other not. In this case, *Kpn*I/*Mse*I digestion produces a short *Kpn*I/*Mse*I and a *Kpn*I/*Kpn*I fragment, but only a long *Acc65*I/*Mse*I fragment is formed. The remaining 12 permutations [(0000) is trivial] describe fragments where the restriction profile has been altered as a result of genetic and/or epigenetic changes. A full interpretation of all of these codes is given in the form of supplementary information (Figure 1).

Tissue culture-induced event frequency was derived on a per donor basis, by first multiplying the number of *Acc65*I/*Mse*I and *Kpn*I/*Mse*I fragments scored by the number of regenerants (Table 1), to deliver a number of "events". This number was used as a divisor to establish a percentage frequency for each variant pattern. In order to assign the contribution to the overall variance of donor origin and source of explant, these frequencies were arcsin transformed, and ANOVA was performed using an SAS software package [33].

## Authors' contributions

PTB developed the concepts of the methylation sensitive AFLP approach and its theoretical background such as the scoring and quantification of tissue culture events, participated in statistical analysis, and drafted the manuscript. RO prepared plant materials for analysis, carried out the molecular studies and scored gels, and participated in the preparation of data for quantification of the events. RMDK participated in the conceptual work and the drafting of the manuscript. JZ was involved in the concept, the production of the plant material, the design of the tissue culture materials, and participated in writing the manuscript. All the authors have read and approved the final manuscript.

## Figures and Tables

**Table 1:** Source of regenerant lines by donor.

**Donor code**	**'E' regenerants**	**'A' regenerants**	**Total regenerants**
4DH	6	6	12
5DH	10	12	22
8DH	7	12	19
9DH	5	5	10
16DH	9	6	15

total	37	41	78

**Table 2:** Absolute frequencies of recovery of conserved and variant fragments among the regenerants from tissue culture of immature embryo (E) and anther (A) explants.

	4DH		5DH		8DH		9DH		16DH		
AFLP pattern	E	A	E	A	E	A	E	A	E	A	A+E (%)

Not affected by tissue culture											
1111	1761	1746	2966	3234	1806	3077	1570	1529	2952	1966	78.3
1100	274	277	193	230	153	261	257	254	455	306	9.2
0011	102	103	190	222	126	214	113	112	197	127	5.2
Affected by tissue culture											
0001	4	3	5	30	4	15	0	6	0	0	0.2
0111	102	101	30	42	35	62	52	53	19	17	1.8
0101	8	8	0	35	2	6	0	10	0	0	0.2
0100	25	31	26	70	9	18	1	20	0	1	0.7
1011	57	62	48	62	34	66	29	35	44	26	1.6
1010	12	12	0	3	21	36	5	4	0	0	0.3
1001	2	2	0	0	0	0	0	1	4	1	0.0
1000	90	87	31	42	47	89	15	13	28	14	1.6
1110	11	16	15	10	14	22	0	1	1	5	0.3
1101	24	24	6	4	10	10	18	22	8	9	0.5
0010	0	0	0	0	0	0	0	0	0	0	0
0110	0	0	0	0	0	0	0	0	0	0	0

**Table 3:** Analysis of variance with respect to frequency of tissue-culture induced (epi)mutations for explant and donor.

	df	SS	MS	F-ratio	p
Explant	1	0.064	0.064	1.32	0.26 ns
Donor	4	3.835	0.959	19.95	< .0001
					
Explant × Donor	4	0.042	0.010	0.22	0.93 ns
Variation class	3	0.068	0.023	0.15	0.93 ns
					

donor	mean	variation class	mean		

4DH	1.77a	demethylation	1.33a		
8DH	1.38b	de novo methylation	1.27a		
9DH	1.24b	sequence variation	1.24a		
5DH	1.09bc	complex variation	1.22a		
16DH	0.84c				

**Table 4:** Non-randomly behaving (epi)mutations present in > 90% of regenerants but absent from the donor plant (*or vice versa*). A zero value indicates the absence of the particular event.

**AFLP pattern**	**Donor code**				
	
	**4DH**	**5DH**	**8DH**	**9DH**	**16DH**
0001	0	0	0	0	0
0111	17	1	5	9	0
0101	1	0	0	0	0
0100	2	2	1	0	0
1011	8	4	3	5	4
1010	2	0	3	1	0
1001	0	0	0	0	0
1000	15	3	5	3	2
1110	1	3	0	0	0
1101	5	0	0	2	0
0010	0	0	0	0	0
0110	0	0	0	0	0

**Table 5:** Adapters and metAFLP oligomer sequences.

**Adapters/Primers**	**Sequence 5'→3'**
Acc65I adapter 1	CTC GTA GCA TGC GTA CA
Acc65I adapter 2	GTA CTG TAC GCA TGC TAC
KpnI adapter 1	CTC GTA GCA TGC GTA CAG TAC
KpnI adapter 2	ACT CGT ACG ACT GT
MseI adapter 1	TAC TCA GGA CTC ATC
MseI adapter 2	GAG TCC TGA GTA GCA G
Acc65I/KpnI pre-selective primer	GCA TGC GTA CAG TAC C
MseI pre-selective primer	GAT GAG TCC TGA GTA AC
CpG-GAC	CAT GCG TAC AGT ACC GAC
CpG-GCA	CAT GCG TAC AGT ACC GCA
CpG-GGC	CAT GCG TAC AGT ACC GGC
CpXpG-AGG	CAT GCG TAC AGT ACC AGG
CpXpG-AGA	CAT GCG TAC AGT ACC AGA
CpXpG-AGC	CAT GCG TAC AGT ACC AGC
CpXpG-TGC	CAT GCG TAC AGT ACC TGC
MCAA	GAT GAG TCC TGA GTA AC
MCAG	GAT GAG TCC TGA GTA AC
MCAC	GAT GAG TCC TGA GTA AC
MCGC	GAT GAG TCC TGA GTA AC
MCCA	GAT GAG TCC TGA GTA AC
MCCG	GAT GAG TCC TGA GTA AC
